# DNA Methylation Mediates Sperm Quality via *piwil1* and *piwil2* Regulation in Japanese Flounder (*Paralichthys olivaceus*)

**DOI:** 10.3390/ijms25115935

**Published:** 2024-05-29

**Authors:** Wenyu Zong, Yapeng Wang, Lingqun Zhang, Wei Lu, Weigang Li, Fengchi Wang, Jie Cheng

**Affiliations:** 1 MOE Key Laboratory of Marine Genetics and Breeding, College of Marine Life Sciences, Ocean University of China, Qingdao 266003, China; 2 Key Laboratory of Tropical Aquatic Germplasm of Hainan Province, Sanya Oceanographic Institution, Ocean University of China, Sanya 572024, China; 3 Laboratory for Marine Fisheries Science and Food Production Processes, National Laboratory for Marine Science and Technology, Qingdao 266237, China

**Keywords:** DNA methylation, *Paralichthys olivaceus*, *piwi* gene, sperm quality, sex reversal

## Abstract

DNA methylation is an important way to regulate gene expression in eukaryotes. In order to reveal the role of DNA methylation in the regulation of germ cell-specific *piwi* gene expression during spermatogenesis of Japanese flounder (*Paralichthys olivaceus*), the expression profiles of *piwil1* (*piwi-like 1*) and *piwil2* (*piwi-like 2*) genes in the gonads of female, male, and sex-reversed pseudo-male *P. olivaceus* were analyzed, and the dynamic of DNA methylation was investigated. As a result, *piwil1* and *piwil2* genes were highly expressed in the testis of both male and pseudo-male *P. olivaceus*, with significant variation among male individuals. The DNA methylation levels in the promoter regions of both *piwil1* and *piwil2* were negatively correlated with their expression levels, which may contribute to the transcriptional regulation of *piwi* genes during spermatogenesis. There was also sperm quality variation among male *P. olivaceus*, and the sperm curvilinear velocity was positively correlated with the expression of both *piwil1* and *piwil2* genes. These results indicated that the DNA methylation in *piwil1* and *piwil2* promoter regions may affect the initiation of *piwi* gene transcription, thereby regulating gene expression and further affecting the spermatogenesis process and gamete quality in *P. olivaceus*.

## 1. Introduction

Spermatogenesis in teleost species is a complex and highly organized process involving many regulatory factors, and the *piwi* genes are important and involved in the regulation of spermatogonial stem cell proliferation, meiosis, sperm formation, and transposon silencing during spermatogenesis and can maintain the normal morphology of sperm [1]. Specifically, PIWI proteins, belonging to the Argonaute family, can interact with the PIWI-interacting RNAs (piRNAs) to form the PIWI/piRNA complex [2], which is involved in the transposable element silencing and post-transcriptional regulation of genes in germ cells and is of great significance for germ cell development and gametogenesis [3,4,5,6]. In mammals, *piwi* genes are mainly expressed in the testis, which are not only involved in gene regulation and transposon silencing during germ cell differentiation but also crucial for sperm formation [7,8,9]. Among them, *piwil1* is highly expressed in spermatocytes and plays a regulatory role in the translation process at the last stage of spermatogenesis, while *piwil2* is highly expressed in spermatogonial stem cells and plays an important role in the initial stage of spermatogenesis [10]. Moreover, in zebrafish, two *piwi* genes were found: *ziwi* (*piwil1*) and *zili* (*piwil2*), both expressed in male and female gonads [11]. The deletion of *ziwi* and *zili* could lead to the failure of germ cells to enter the differentiation state and eventually cause them to suffer apoptosis [1,12]. In addition, *piwi* genes have also been confirmed to be highly expressed in gonads, especially in the testis, and are involved in the regulation of the reproductive cycle in teleost fish such as Japanese medaka (*Oryzias latipes*) [13], pufferfish (*Takifugu fasciatus*) [14], Nile tilapia (*Oreochromis niloticus*) [15], and turbot (*Scophthalmus maximus*) [16]. Especially in Chinese tongue sole (*Cynoglossus semilaevis*) with sexual reversal scenario from female to pseudo-male, *piwil2* is located on the Z chromosome, and the expression of *piwil2* in the gonads is the highest in male, followed by pseudo-male, and the lowest in female, which plays an important role in spermatogenesis [17].

The development of germ cells is controlled by a dynamic interplay between extrinsic signaling pathways and regulations at epigenetic, transcriptional, and posttranscriptional levels. DNA methylation is one of the major epigenetic regulation patterns for gene expression and plays an essential role in gene silencing, majorly functioning in the gene promoter region [18,19,20,21]. Many studies have revealed that DNA methylation-mediated transcriptional regulation affects teleost sex differentiation and gametogenesis by controlling the expression of specific genes. For example, DNA methylation in the aromatase *cyp19a1a* gene promoter could regulate its transcriptional activity and contribute to sexual development in ricefield eel (*Monopterus albus*) [22], European sea bass (*Dicentrarchus labrax*) [23], loach (*Misgurnus anguillicaudatus*) [24], and Chinese tongue sole (*C. semilaevis*) [25]. Methylation of the CpG island in the *sox9a* promoter leads to differential expression in the testis and ovary of the topmouth culter (*Culter alburnus*) [26]. In addition, hypermethylation of the CpG island in the promoters of *piwil1* and *piwil2* leads to gene silencing, which can induce primary seminoma and testicular germ cell tumors in mammals and even lead to male sterility [27,28].

Japanese flounder (*Paralichthys olivaceus*) is an important marine-cultured fish in North Asia that has significant sex dimorphism with bigger females [29]. However, *P. olivaceus* is susceptible to environmental factors such as temperature during the key sex differentiation period, resulting in sex reversal of the genetic female into pseudo-males [30]. Therefore, the problems of imbalanced sex ratio and reduced gamete quality affect the breeding process, and high-quality *P. olivaceus* gametes are urgently needed. Our previous studies have identified the male-specific piRNA genes in *P. olivaceus*, including *piwil1*/*piwil2* [31] and *tdrd* genes [32,33], with the DNA methylation contributing to the expression difference between males and females [34]. However, the regulatory differences in the expression of *piwil* genes between male and pseudo-male *P. olivaceus* and the relationship between *piwil* genes and sperm quality are still unclear. Therefore, with the recently identified *P. olivaceus* sex-determining gene *amhy* [35], in this study, the *P. olivaceus* genetic sex was identified between male and pseudo-male, and the expression regulation of *piwi* genes and their correlation with sperm quality were investigated. The results will enrich the theoretical basis for the reproductive physiology of *P. olivaceus* and provide information for further exploring the molecular mechanism of reproductive regulation in teleost fish.

## 2. Results and Discussion

### 2.1. Identification of Genetic Sex in P. olivaceus

**Figure 1:**
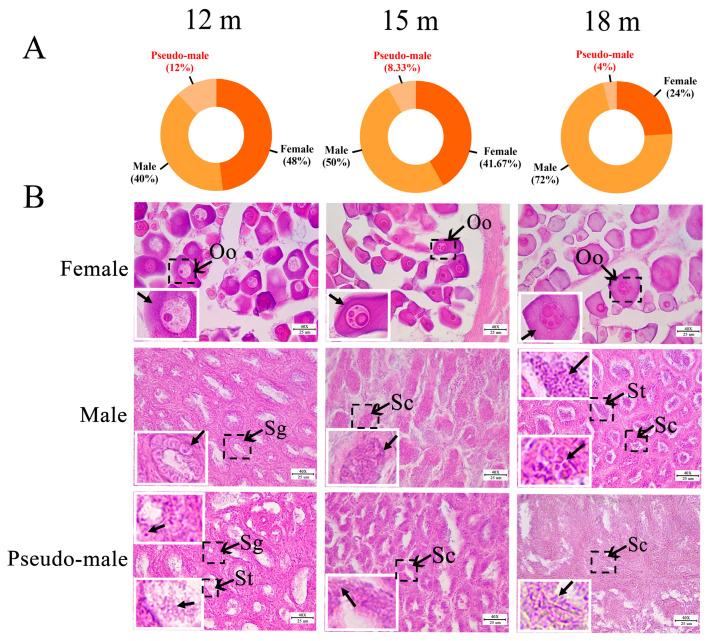
Sex ratio and gonad histology of *P. olivaceus*. (**A**) Sex ratio of *P. olivaceus* at different developmental stages (12 m, 15 m, and 18 m); (**B**) Gonad histology of *P. olivaceus* at different developmental stages (12 m, 15 m, and 18 m). Oo: Oocyte; Sg: Spermatogonia; Sc: Spermatocyte; St: Spermatid. Scale bar: 25 µm. The white frame is an enlarged view of the arrowed black dashed box.

According to the PCR of the *amhy* sex marker (Figure S1A) and gonad histological examination, the genetic and phenotypic sex of *P. olivaceus* was identified. Among the three sampled populations, 12 females (48%), 10 males (40%), and 3 sex reversed pseudo-males (12%) were identified in 12-month-old *P. olivaceus*; 10 females (41.67%), 12 males (50%), and 2 pseudo-males (8.33%) were from 15-month-old *P. olivaceus*; and 6 females (24%), 18 males (72%), and 1 pseudo-male (4%) were from 18-month-old *P. olivaceus* (Figure 1A). The proportion of males in the three groups was higher than females, of which the proportion of pseudo-males was generally low (4–12%, Figure 1A). The proportion of pseudo-males was similar to that in the phylogenetically related *C. semilaevis*; among 45 specimens caught from the sea, 20 *C. semilaevis* were males (44.4%), 4 were pseudo-males (8.89%), and 21 were females (46.67%) [36]. In addition, the total length and body weight of *P. olivaceus* increased gradually from 12 months (30.8 ± 3.5 cm, 312.3 ± 59.2 g) to 15 months (31.1 ± 1.6 cm, 318.5 ± 48.4 g), and further to 18 months (38.8 ± 1.9 cm, 550.4 ± 90.7 g) (Figure S1B). The growth of females was slightly faster than that of males and pseudo-males, while no significant difference was found between males and pseudo-males (Figure S1B), which was consistent with the sex dimorphism of growth in *P. olivaceus*. Similarly, the growth rate of females was also significantly faster than that of males and pseudo-males in *C. semilaevis* [37,38]. Moreover, as the sex marker, the *amh* gene was significantly higher expressed in *P. olivaceus* testis than in ovaries (Figure S1C), which agreed with its male-specific function that regulates spermatogenesis and promotes male sex differentiation and the development and maintenance of gonads in teleosts [39,40].

### 2.2. Gonadal Histology of P. olivaceus

Through hematoxylin and eosin (H&E) staining, gonad tissues were examined for the three sampled *P. olivaceus* groups. The female ovaries mainly contained oocytes with lipid droplets in the cytoplasm, and the diameter of the oocytes gradually increased along with development (Figure 1B). The 12-month-old *P. olivaceus* testis was mainly composed of spermatogonia and was in the early stage of spermatogenesis (Figure 1B). At 15 months old, the germ cells were mainly spermatocytes, which were smaller than spermatogonia (Figure 1B). There were a large number of spermatids in the testes of 18-month-old males, and the testis was in the late stage of spermatogenesis (Figure 1B). In addition, there was no significant difference in the development of testes between males and pseudo-males at the same stage (Figure 1B). The testis of the 18-month-old pseudo-male was poorly developed, and no sperm cells were found, mainly as spermatocytes (Figure 1B). However, it has been found that the testes of 12-month-old triploid pseudo-male *C. semilaevis* are significantly hindered compared with those of the normal male, with a large cavity in the spermatic lobule regions, no spermatocytes or mature sperm, and almost no spermatocytes in the triploid *C. semilaevis* [41]. Due to the limited proportion of pseudo-males identified here, whether there are differences in the developmental status between pseudo-males and males in *P. olivaceus* warrants further investigation.

### 2.3. Expression Profile of piwil1 and piwil2 Genes in P. olivaceus Gonads

**Figure 2:**
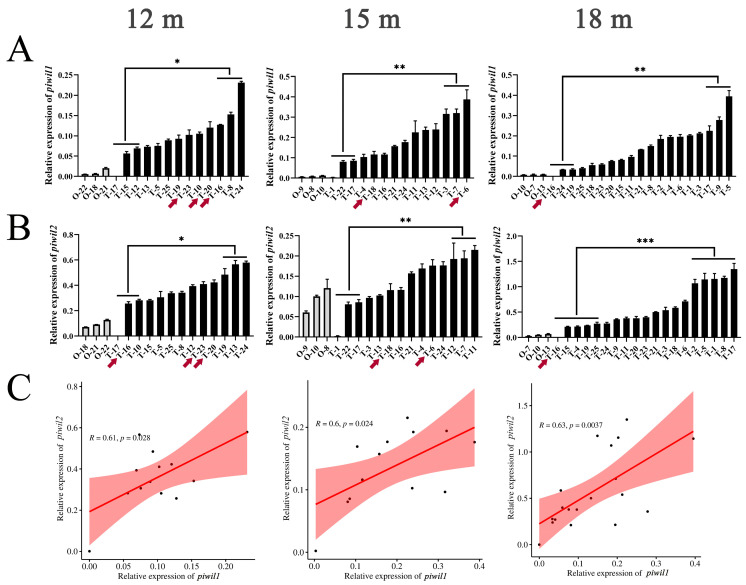
Expression profiles of *piwil1* and *piwil2* genes in *P. olivaceus* gonads. (**A**) Expression of *piwil1* in *P. olivaceus* gonads (12 m, 15 m, and 18 m); (**B**) Expression of *piwil2* in *P. olivaceus* gonads (12 m, 15 m, and 18 m); (**C**) Pearson coefficient correlation of the expression levels between *piwil1* and *piwil2* in three groups of *P. olivaceus* testes (12 m, 15 m, and 18 m). The red arrow indicates the pseudo-males. * represents *p* < 0.05; ** represents *p* < 0.01; *** represents *p* < 0.001.

The expression of *piwil1* and *piwil2* genes in *P. olivaceus* gonads was characterized. As expected, *piwil1* and *piwil2* were preferentially expressed in *P. olivaceus* testes than in ovaries (Figure 2A,B). It is worth noting that the expression of *piwil1* and *piwil2* in the testes was not different between males and pseudo-males, but there were significant differences between the higher and lower parts of males in all three groups (Figure 2A,B). However, in *C. semilaevis*, the expression of *piwil2* is different in the testes of males and pseudo-males; it is higher in normal males than in pseudo-males [17]. In addition, there was a significant positive correlation between the expression of *piwil1* and *piwil2* (*p* < 0.05) in *P. olivaceus* testes, indicating that they may play a coordinated function together in testes (Figure 2C). In mammals, *hiwi* (*piwil1*) expression continues from spermatocyte to late gametogenesis, while *hili* (*piwil2*) mainly functions in early spermatogenesis [10]. In zebrafish, *ziwi* (*piwil1*) is expressed in both spermatogonium and spermatocyte [11], and *zili* (*piwil2*) is present predominantly in the cytoplasm of all stages of spermatogenesis, except the fully differentiated sperm [12]. However, how *piwil1* and *piwil2* may synergically regulate gametogenesis is still unclear, and further investigation is needed.

### 2.4. DNA Methylation Dynamics of piwi Genes in P. olivaceus Gonads

**Figure 3:**
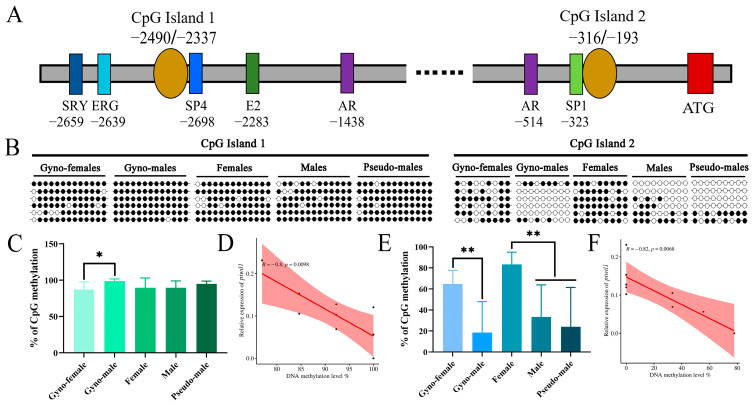
DNA methylation profiles of the *piwil1* promoter region in the gonads of *P. olivaceus*. (**A**) The predicted CpG islands and transcription factors in the *piwil1* promoter region; (**B**) A schematic representation of *piwil1* promoter CpG methylation in *P. olivaceus* gonads. The black circle represents the methylated site, and the white circle represents the unmethylated site. Each row represents a monoclonal sequence from one individual in each group (n = 6); (**C**) The average methylation level in the first CpG island of *piwil1* promoter in different gonads; (**D**) The linear regression between the first CpG island DNA methylation levels and expression levels of *piwil1* in *P. olivaceus*. (**E**) The average methylation level in the second CpG island of *piwil1* promoter in different gonads; (**F**) The linear regression between the second CpG island DNA methylation levels and expression levels of *piwil1* in *P. olivaceus*. * represents *p* < 0.05; ** represents *p* < 0.01.

Two CpG islands were predicted in the promoter region of *P. olivaceus piwil1* gene (Figure S2A), and the first CpG island was located in the ATG upstream region (−2490 bp to −2337 bp) with a length of 154 bp; the second CpG island was located at −316 bp to −193 bp with 124 bp in length (Figure 3A). The putative transcription factor binding sites were predicted, including one Y chromosome sex-determining region (SRY), one ETS-related gene binding site (ERG), one SP4 binding site, and one estrogen binding site (E2), two androgen receptor binding sites (AR), and one SP1 binding site, with the SP4 and SP1 sites near the two CpG islands (Figure 3A). The DNA methylation level of the first CpG island in the *piwil1* promoter region was characterized by gynogenetic *P. olivaceus* females and pseudo-males, as well as the 12-month-old *P. olivaceus* and all pseudo-males from all three stages. As a result, the methylation levels of the first CpG island in gynogenetic female ovaries (87.18%), gynogenetic pseudo-male testes (98.72%), female ovaries (89.74%), male testes (89.74%), and pseudo-male testes (94.87%) were generally similar and high (Figure 3B,C). The methylation level was only significantly different between the testes and ovaries of gynogenetic *P. olivaceus* (*p* < 0.05), which was higher in testes than in ovaries and was in contrast to the highly expression of *piwil1* in the testes (Figure 3C). Subsequently, the methylation level of the second CpG island from *piwil1* was analyzed, with the gynogenetic pseudo-male testes (18.52%) being significantly lower than that of the gynogenetic female ovaries (64.81%) (Figure 3E). Most of the 9 CpG sites detected in gynogenetic pseudo-males were not methylated, with only two gynogenetic pseudo-males being similar to gynogenetic females (Figure 3B). Similarly, the methylation level in the testes of male (33.33%) and pseudo-male (24.07%) *P. olivaceus* was similar and significantly lower than that of female (83.33%) (Figure 3E). Therefore, the methylation level in male and pseudo-male testes was relatively similar and low, which agreed with the highly *piwil1* expression in both male and pseudo-male testes (Figure 3B,E). In addition, the methylation level of *piwil1* exhibited a significantly negative correlation with its expression level in testes in both the first CpG island (R = −0.8, *p* < 0.05) (Figure 3D) and the second CpG island (R = −0.82, *p* < 0.05) (Figure 3F), indicating that the DNA methylation may mediate the *piwil1* expression in testes of both male and pseudo-male *P. olivaceus*.

**Figure 4:**
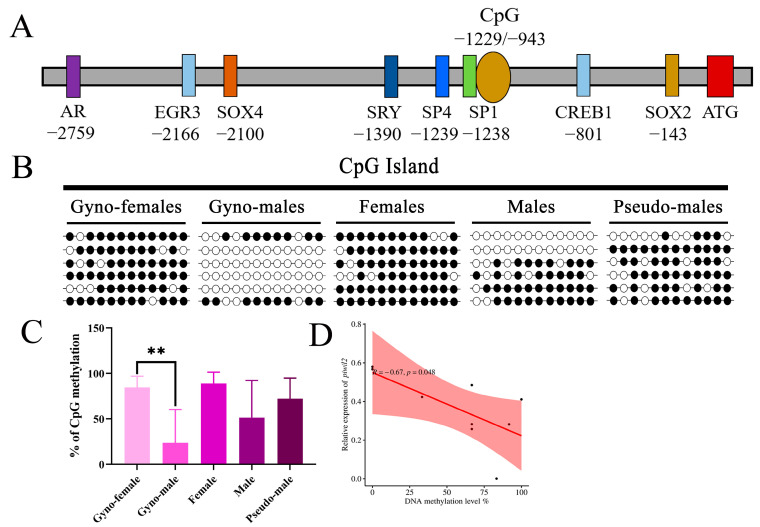
DNA methylation profiles of the *piwil2* promoter region in the gonads of *P. olivaceus*. (**A**) The predicted CpG islands and transcription factors in the *piwil2* promoter region; (**B**) A schematic representation of *piwil2* promoter CpG methylation in *P. olivaceus* gonads. The black circle represents the methylated site, and the white circle represents the unmethylated site. Each row represents a monoclonal sequence from one individual in each group (n = 6); (**C**) The average methylation level of *piwil2* promoter in different gonads; (**D**) The linear regression between DNA methylation levels and expression levels of *piwil2* in *P. olivaceus*. ** represents *p* < 0.01.

In addition, one single CpG island was predicted in the promoter region of *piwil2* (Figure S2B), which was 287 bp in length and located in the upstream of ATG from −1229 bp to −943 bp (Figure 4A). The putative transcription factor binding sites were predicted, including AR, EGR3, SOX4, SRY, SP4, SP1, CREB1, and SOX2 binding sites, with SP1 (GC box) located near the CpG island (Figure 4A). The DNA methylation level of the *piwil2* promoter region in the testes of gynogenetic pseudo-males (23.61%) was significantly lower than that in the gynogenetic female ovaries (84.72%), with the 12 CpG sites being unmethylated in most gynogenetic pseudo-males and only two gynogenetic pseudo-males presenting a similar methylation pattern to gynogenetic females (Figure 4B). In addition, the methylation level of the female ovaries (88.89%) in common *P. olivaceus* was also higher than that of the testes in pseudo-males (72.22%) and males (51.39%), with the methylation level of pseudo-males between males and females (Figure 4C). This result was similar to the methylation pattern of *piwil2* in the gonads of *C. semilaevis* [17], with hypermethylation of the ovary being observed, followed by pseudo-male testis, and lowest in male testis. Moreover, the methylation level of *piwil2* also exhibited a significantly negative correlation with its expression level in the testes (R = −0.67, *p* < 0.05) (Figure 4D), indicating that the DNA methylation contributed to the expression regulation of *piwil2* in *P. olivaceus* gonads. In addition, transcription factor SP1 binding sites were found near the second CpG island of *piwil1* (Figure 3A) and the CpG island of *piwil2* (Figure 4A). Therefore, the methylation status at the site of transcription factor SP1 may affect both *piwil1* and *piwil2* gene expression in *P. olivaceus* gonads, which warrants further experimental confirmation.

### 2.5. DNA Methylation Profile of piwi Genes at Different Stages

**Figure 5:**
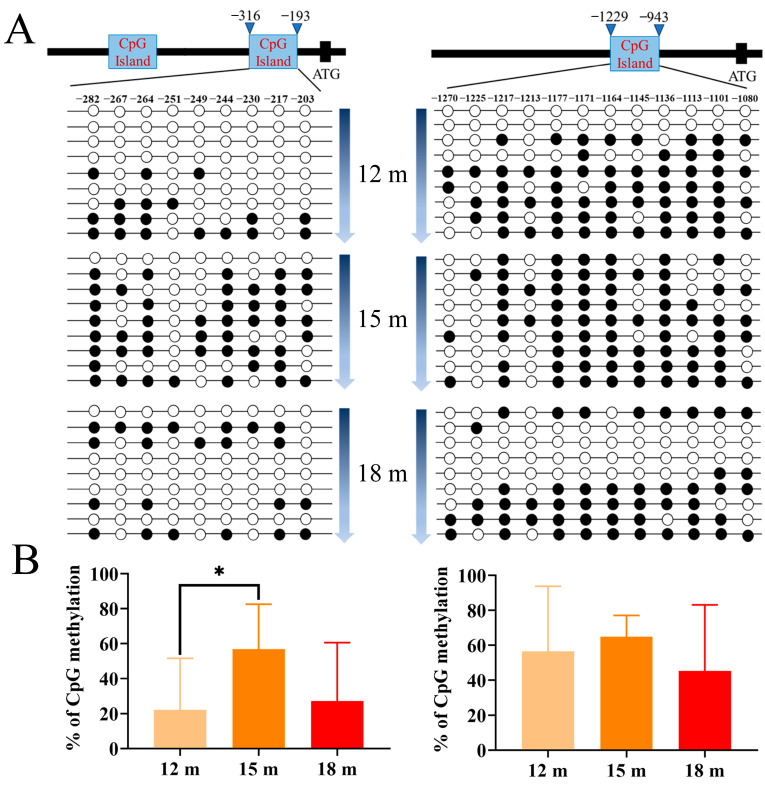
DNA methylation profiles of *piwi* genes in *P. olivaceus* testis at different developmental stages. (**A**) Schematic representation of different CpG methylation of *piwil1* (**left**) and *piwil2* (**right**) in testes at different developmental stages. The black circle represents the methylation site, and the white circle represents the unmethylated site. Each row represents a monoclonal sequence from one individual of each group (n = 9) with the descending gene expression level; (**B**) the average methylation level variations at different stages. * represents *p* < 0.05.

We further detected the DNA methylation level of the second CpG island in *piwil1* and the CpG island of *piwil2* in *P. olivaceus* gonads at different developmental stages. Nine testes were selected at each stage, including all pseudo-males detected. As a result, the *piwil1* methylation level of the 12-month-old *P. olivaceus* was the lowest (22.22%), followed by 18-month-old *P. olivaceus* (27.16%), and 15-month-old *P. olivaceus* (56.79%) (Figure 5A,B). For *piwil2*, the DNA methylation levels were 56.48% (12 m), 64.81% (15 m), and 45.37% (18 m), respectively (Figure 5A,B). Interestingly, the average methylation level of the second CpG island in *piwil1* increased from 12-months to 15-months, and then declined from 15-months to 18-months (Figure 5A,B), which may be related to the spermatogenesis process from spermatocyte to spermid as shown in Figure 1B. The average methylation level of the CpG island in *piwil2* promoter was relatively similar in different stages (Figure 5A,B), indicating the putative functional divergence between *piwil1* and *piwil2*. In *M. anguillicaudatus*, the average methylation level of *cyp19a1a* had no significant change along the developmental stages of the testis but showed a significant difference along the developmental stages of the ovary, which was related to *cyp19a1a* expression in different stages [24].

### 2.6. Characterization of P. olivaceus Sperm Quality with piwi Expression

**Figure 6:**
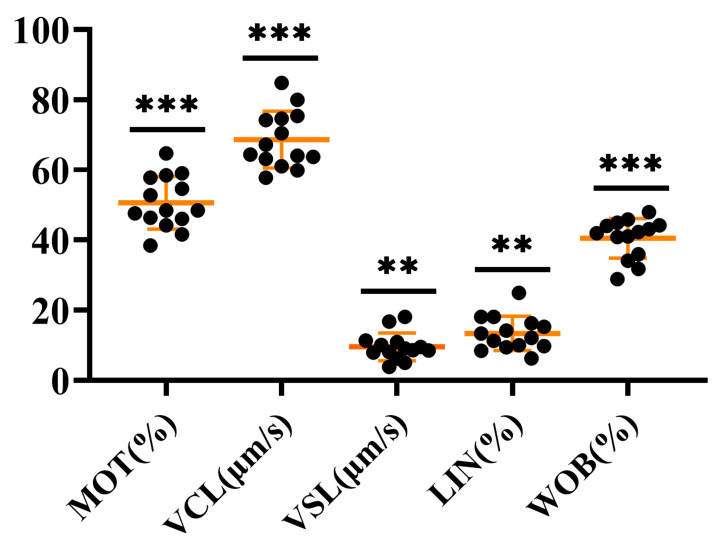
Sperm quality in *P. olivaceus* with different indicators. ** represents *p* < 0.01 and *** represents *p* < 0.001 between the highest three and lowest three individuals for each quality indicator (Table S1).

The quality of *P. olivaceus* semen samples was tested by the SCA sperm analyzer, including sperm motility (MOT), curvilinear velocity (VCL), straight line velocity (VSL), linearity (LIN), and wobble index (WOB) (Figure 6 and Table S1). Sperm motility is usually measured by the fecundity index MOT, and normal sperm motility is 40% or more active sperm per milliliter of semen [42]. Here, only the MOT of S15 individuals was below 40%, and the MOT of the remaining individuals ranged from 41.63% to 64.67% (Figure 6 and Table S1). VCL and VSL are also important parameters to reflect sperm motility, and the VCL (57.76–84.82 μm/s) and VSL (3.90–18.11 μm/s) of all semen samples were significantly diversified (Figure 6 and Table S1). Moreover, LIN refers to the straight-line separation degree of the sperm motion curve, namely VSL/VCL, ranging from 6.31 to 24.96% (Figure 6 and Table S1), and the swing index refers to the swing scale of the sperm head along the actual trajectory in space, ranging from 28.91% to 47.97% (Figure 6 and Table S1). The significant differences among the indicators suggested the diverse sperm quality in *P. olivaceus* testes (Figure 6 and Table S1). There were also significant differences in the quality of sperm produced by males and pseudo-males in *C. semilaevis*, with the ZW-ZZ sex determination, and after W-sperm from pseudo-male fertilized eggs, WW-type superfemale should be produced theoretically, but no superfemale was found in adult fish in relevant experiments [25]. Therefore, the study speculated that the W-type sperm produced by pseudo-males may be defective, or the F1 generation individuals produced may have a higher mortality rate during development [43,44].

**Figure 7:**
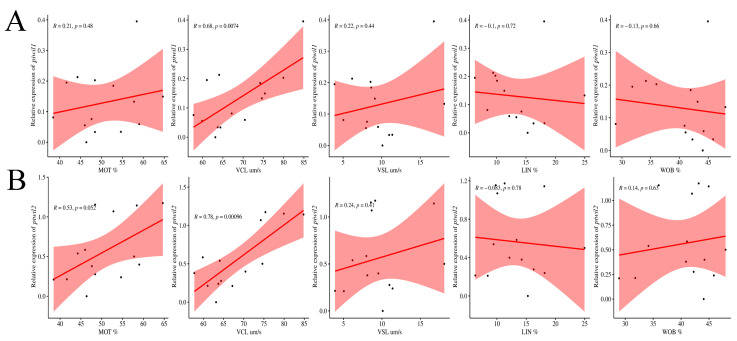
Pearson coefficient correlation between sperm quality and *piwi* gene expression profile in *P. olivaceus*. (**A**) Coefficient correlation between sperm quality and *piwil1* expression profile; (**B**) Coefficient correlation between sperm quality and *piwil2* expression profile.

Interestingly, VCL has a strong positive correlation with the expression of *piwil1* (R = 0.68, *p* < 0.05) and *piwil2* (R = 0.78, *p* < 0.05) (Figure 7), as the higher the *piwi* expression, the faster the VCL. Moreover, there was also a weak positive correlation between MOT and *piwil2* expression (R = 0.53, *p* = 0.052), which may need further verification in other populations (Figure 7). Generally, the fast movement speed represents the strong activity of sperm, indicating that the *piwi* gene expression may improve sperm activity and contribute to the fertilization capacity of sperm, as well as the reproduction of offspring. Recently, sperm motility has also been considered one of the most useful markers of spermatozoa quality in fish [45]. In zebrafish, ectopic expression of miRNA significantly reduced motility traits, namely VCL, VSL, and average path velocity (VAP), and the fertilization capacity of spermatozoa [46]. In the sperm of Senegalese sole (*Solea senegalensis*), there are significant differences in VCL after sperm activation. A similar, albeit not significant, tendency was found for VSL and VAP [47]. Sperm DNA methylation status may also be related to sperm quality assessment. In this case, alterations in DNA methylation have also been associated with male infertility in humans [48] and fish [49]. Therefore, the reason for the difference in *P. olivaceus* sperm quality is not yet clear, and whether it is related to the DNA methylation degree of the *piwi* gene needs to be further verified.

## 3. Materials and Methods

### 3.1. Material Statement

The normal *P. olivaceus* specimens (12-, 15-, and 18-month-old) used in the experiments were obtained from Xiaogang Wharf in Qingdao, Shandong Province, China, with a similar genetic background. According to Sun (2008) [50], the spermatogenesis of *P. olivaceus* was asynchronized, and the mature sperm could be identified at the age of 12 months. The gynogenetic *P. olivaceus* females and sex-reversed pseudo-males (18-month-old) were induced by ultraviolet irradiation and hydrostatic pressure treatment in our previous study [51]. Gonad tissues were sampled, part of which was frozen in liquid nitrogen and stored at a −80 °C refrigerator for subsequent DNA and RNA extraction, and the other part was added to 4% paraformaldehyde (PFA) for subsequent tissue sections. In addition, semen was collected from the 18-month-old male *P. olivaceus* for sperm quality analysis.

### 3.2. DNA Isolation and PCR

The gonad tissues of *P. olivaceus* were fully lysed with TNES lysis buffer and protease K, and DNA was extracted by phenol/chloroform. Three groups of *P. olivaceus* at different stages were subjected to PCR using DNA as a template with the *amhy* primers listed in Table S2 [35]. The PCR process was as follows: 95 °C for 5 min, followed by 35 cycles at 95 °C for 30 s, 58 °C for 30 s, 72 °C for 1 min, and 72 °C for 5 min. After PCR amplification, the products were subjected to agarose gel electrophoresis. The PCR product with one band (586 bp) was from the genetic female, and two bands (586 bp and 312 bp) were from the genetic male [35]. Together with the gonad histological observation, female, male, and pseudo-male *P. olivaceus* were identified.

### 3.3. Histological Examination of P. olivaceus Gonads

H&E staining with the gonad tissues of *P. olivaceus* was performed as described previously [32]. The 4% PFA-fixed testes were treated with a methanol gradient (30%, 50%, 70%, 80%, 90%, 95%, 100%). The tissue was completely dehydrated by anhydrous ethanol treatment, and became transparent by xylene treatment. After the tissue treated with paraffin and xylene at 1:1 for 1 h, it was embedded in paraffin twice for 1.5 h. Then, the tissues were sliced with a microtome, and the slice thickness was 5 μm. After drying water at 37 °C and then stained according to the following procedure: apply xylene infiltration 2 times, xylene and ethanol (1:1) mixed, anhydrous ethanol 2 times, 5 min per step; then apply a 95%, 80%, 70%, 50%, 30% anhydrous ethanol gradient, distilled water twice, each step for 2 min; hematoxylin staining for 10 s; then reverse the above experimental steps; perform eosin staining for 6 s after a 95% ethanol gradient; use a neutral gum seal; and observe with a microscope (SMZ-B6).

### 3.4. Total RNA Isolation and qRT-PCR

*P. olivaceus* gonad total RNA was extracted using TRIzol^®^ reagent (Sparkjade, Jinan, China) and purified by the Recombiant DNase I (AG, Changsha, China) and RNA Cleaning Kit (Biomed, Beijing, China). The concentration and integrity of RNA were examined by electrophoresis and the NanoPhotometer Pearl 360 (Implen, Munich, Germany). The gonadal RNA was reverse transcribed using the All-In-One 5 × RT Mast Mix kit (Abm, Zhenjiang, China). The cDNA samples were subsequently used as templates for qRT-PCR experiments with *piwil1* and *piwil2*. All gene-specific primers were designed using the IDT website (Table S2). *ACTB* and *UBCE* were used as internal controls, and the samples were repeated in triplicate. qRT-PCR was conducted with three females, all males, and pseudo-males of the three sampled groups (12 m, 15 m, and 18 m). The total volume of the reaction was 20 µL, containing 2 µL template cDNA (5 ng/µL), 0.4 µL each forward/reverse primers (10 µmol/L), 10 µL SYBR^®^FAST qPCR Master Mix (2×), and 7.2 µL of nuclease-free water. The qRT-PCR process was as follows: 95 °C for 30 s, followed by 40 cycles at 95 °C for 5 s and 58 °C for 30 s. The melting curves and PCR amplification efficiency for each primer are shown in Figure S3 and Table S3. Relative expression levels were calculated by the 2^−∆∆Ct^ method. The significance between the two groups was analyzed by *t*-test. The results were graphed using Prism software (GraphPad Prism 8.0.2).

### 3.5. Prediction of CpG Islands

The promoter region of *piwi* genes was 3000 bp upstream of the default transcription start site. The sequence information of the promoter region of *piwil1* and *piwil2* was obtained through the NCBI (https://www.ncbi.nlm.nih.gov/, accessed on 15 May 2023). The CpG island in the promoter regions was predicted by the MethPrimer website (http://www.urogene.org/methprimer/, accessed on 16 May 2023), and the bisulfite sequencing PCR (BSP) methylation primers were designed in the CpG island region (Table S2).

### 3.6. Bisulfite Modification Sequencing

The gonad DNA of *P. olivaceus* was treated with bisulfite using the EZ DNA Methylation-GoldTM Kit (Zymo Research, Orange, CA, USA). The bisulfite-treated DNA was used as a template, and the methylation primers of the CpG island in the promoter regions of *piwil1* and *piwil2* were used to amplify them. The PCR process was as follows: 95 °C for 5 min, followed by 35 cycles at 95 °C for 30 s, 50 °C (*piwil1*)/58 °C (*piwil2*) for 30 s, 72 °C for 1 min, and 72 °C for 5 min. The amplified product was recovered by the gel recovery kit (CWBIO, Beijing, China), ligated to the pMDTM 19-T cloning vector (TaKaRa, Dalian, China), and transformed into *E. coli* (DH5α). After being cultured at 37 °C for 12 h on a cell culture plate containing penicillin resistance, the positive clones (Table S4) were selected for sequencing at Sangon Biotech (Shanghai, China). BiQ-Analysis software (v2.02) was used to compare the sequencing results before and after bisulfite modification and generate methylation maps. The percentage of methylation level was calculated according to the ratio of the number of CpG sites with DNA methylation to the total number of CpG sites detected.

### 3.7. Prediction of Transcription Factor Binding Sites in the Promoter Region

The hTFtarget online website (http://bioinfo.life.hust.edu.cn/hTFtarget, accessed on 6 September 2023) was used to predict the transcription factor binding sites in the promoter regions of *piwil1* and *piwil2* genes, and the Score > 10 was set.

### 3.8. Sperm Quality Analysis of P. olivaceus

The semen samples were taken from the 18-month-old *P. olivaceus* males. After cutting the vertebra with scissors, the whole testis was taken out of the abdomen of the fish through the cloacal hole, and the semen was sampled from the sperm duct with a pipette. The semen was temporarily stored in a sterile 1.5 mL centrifuge tube and placed on ice to prevent the effect of high temperatures on sperm motility. A total of 19 male fish were sampled, of which 14 were semen, and 5 male fish were not found semen in the vas deferens, so no semen samples were taken. After the fresh semen was activated with seawater, 3 μL was added to the groove of the disposable sperm detection plate, and the sperm motility (MOT), curve velocity (VCL), linear velocity (VSL), linearity (LIN), and wobble index (WOB) were analyzed by the SCA sperm class analyzer (SCA 6.4.0.82, Microptic, Barcelona, Spain) to determine the quality of sperm with a positive phase-contrast microscope (Nikon ECLIPSE E200, Tokyo, Japan).

### 3.9. Pearson Coefficient Correlation Analysis

Pearson coefficient correlation analysis was conducted using online tools (http://www.bioinformatics.com.cn/, accessed on 19 February 2024). When *p* < 0.05, it was considered that there was a significant correlation; 0 < R < 1 was a positive correlation, and −1 < R < 0 was a negative correlation.

## 4. Conclusions

In summary, no difference was identified in the expression of *piwil1* and *piwil2* between the testes of male and pseudo-male *P. olivaceus*, as well as in the DNA methylation levels. However, the expression of *piwil1* and *piwil2* in *P. olivaceus* testes represents remarkable individual variation, which may be mediated by the DNA methylation in the gene promoter region to affect the binding of transcription factors to the promoter and then the transcription initiation, thereby regulating the *piwi* gene expression. The expression of *piwil1* and *piwil2* is also correlated with sperm quality, which provides novel potential for them as gamete quality biomarkers. These results enrich the theoretical basis of the reproduction physiology of *P. olivaceus* and have important significance for the basic theory and practice of reproductive regulation in *P. olivaceus*.

## Data Availability

The datasets generated from this study can be found in the online Supplementary Materials.

## Supplementary Materials

The following supporting information can be downloaded at: https://www.mdpi.com/article/10.3390/ijms25115935/s1.
