# Supplementary material for: DNA Methylation Mediates Sperm Quality via piwil1 and piwil2 Regulation in Japanese Flounder (Paralichthys olivaceus)
Source: Int J Mol Sci. 2024 May 29;25(11):5935. doi: 10.3390/ijms25115935 (PMC11172970; doi:10.3390/ijms25115935)
Supplement: Supplementary file 1 [file ijms-25-05935-s001.zip › Table S1. Sperm quality.pdf]

Supplementary Table S1. Statistics of the sperm quality test results of *P.olivaceus*.

	MOT (%)	VCL (μm/s)	VSL (μm/s)	LIN (%)	WOB (%)
S1	48.42	79.93	8.56	9.8	35.99
**S2**	52.81	74.22	8.66	10.07	41.95
S3	44.24	64.07	6.17	9.42	34.16
S4	41.63	61.05	3.90	6.31	31.80
S5	58.42	84.82	16.76	18.09	44.94
S8	64.67	75.37	9.12	11.31	43.15
S15	38.45	67.18	5.04	8.43	28.91
S16	46.37	63.14	10.10	15.28	44.05
**S18**	46.01	59.85	7.97	13.36	41.06
S19	54.63	63.71	11.38	18.14	45.86
S20	47.62	57.76	8.10	14.23	40.88
S21	57.78	74.62	18.11	24.96	47.97
S23	59.00	70.43	9.53	12.15	44.19
**S24**	48.42	64.38	10.97	16.28	42.25

The red background indicates the three highest values in each index, while the blue background indicates the three lowest values in each index.
