# Supplementary material for: DNA Methylation Mediates Sperm Quality via piwil1 and piwil2 Regulation in Japanese Flounder (Paralichthys olivaceus)
Source: Int J Mol Sci. 2024 May 29;25(11):5935. doi: 10.3390/ijms25115935 (PMC11172970; doi:10.3390/ijms25115935)
Supplement: Supplementary file 1 [file ijms-25-05935-s001.zip › Table S2. Primers.pdf]

Supplementary Table S2. List of primers used in the experiment.

Primer name	Primer sequence (5’ – 3’)	purpose	Product length
*Pol_amh*_PCR_Fw	AAGTTCAGTTCAGTTGCACAGC	PCR	563 bp
*Pol_amh*_PCR_Rv	CTTGACAAACAGGGCATGAATA	PCR	
*Pol_amh*_qPCR_Fw	AGCTCTGGAGTTGACCTTTG	qRT-PCR	104 bp
*Pol_amh*_qPCR_Rv	CGTTCAGGTCGTCACCTTTA	qRT-PCR	
*Pol_piwil1*_qPCR_Fw	GATTGGACGCAACTACTATAACCC	qRT-PCR	160 bp
*Pol_piwil1*_qPCR_Rv	AAGGACTGTCTCACTACGAAGCAC	qRT-PCR	
*Pol_piwil2*_qPCR_Fw	AGGCTTTATCAGACATCTCCCACG	qRT-PCR	188 bp
*Pol_piwil2*_qPCR_Rv	CCCATGTAACTCTACAGGCAGTCA	qRT-PCR	
*Pol_ACTB*_qPCR_Fw	CCACCGCAAATGCTTCTA	qRT-PCR	204 bp
*Pol_ACTB*_qPCR_Rv	ACTGTCTCCATCGTTCCA	qRT-PCR	
*Pol_UBCE*_qPCR_Fw	TTACTGTCCATTTCCCCACTGAC	qRT-PCR	127 bp
*Pol_UBCE*_qPCR_Rv	GACCACTGCGACCTCAAGATG	qRT-PCR	
*Pol_piwil1*_M1_Fw	TTGTGAAATTGGAGTTAGTTTGATATATTA	Bisulfite sequencing PCR	185 bp
*Pol_piwil1*_M1_Rv	AAAAAAAATACACCCTACCAAAAAC	Bisulfite sequencing PCR	
*Pol_piwil1*_M2_Fw	GAGTTGTTAGTATGTTAGTTGAAGATAG	Bisulfite sequencing PCR	178 bp
*Pol_piwil1*_M2_Rv	ATATTAAAAAATTATAACACAAATACATCA	Bisulfite sequencing PCR	
*Pol_piwil2*_M_Fw	GGTGAAGTTGTTTTTTAGTGAGTTG	Bisulfite sequencing PCR	285 bp
*Pol_piwil2*_M_Rv	AAAAACCTCTCAAACAATTTCCTAC	Bisulfite sequencing PCR	
