# Supplementary figures and images for: DNA Methylation Mediates Sperm Quality via piwil1 and piwil2 Regulation in Japanese Flounder (Paralichthys olivaceus)
Source: Int J Mol Sci. 2024 May 29;25(11):5935. doi: 10.3390/ijms25115935 (PMC11172970; doi:10.3390/ijms25115935)

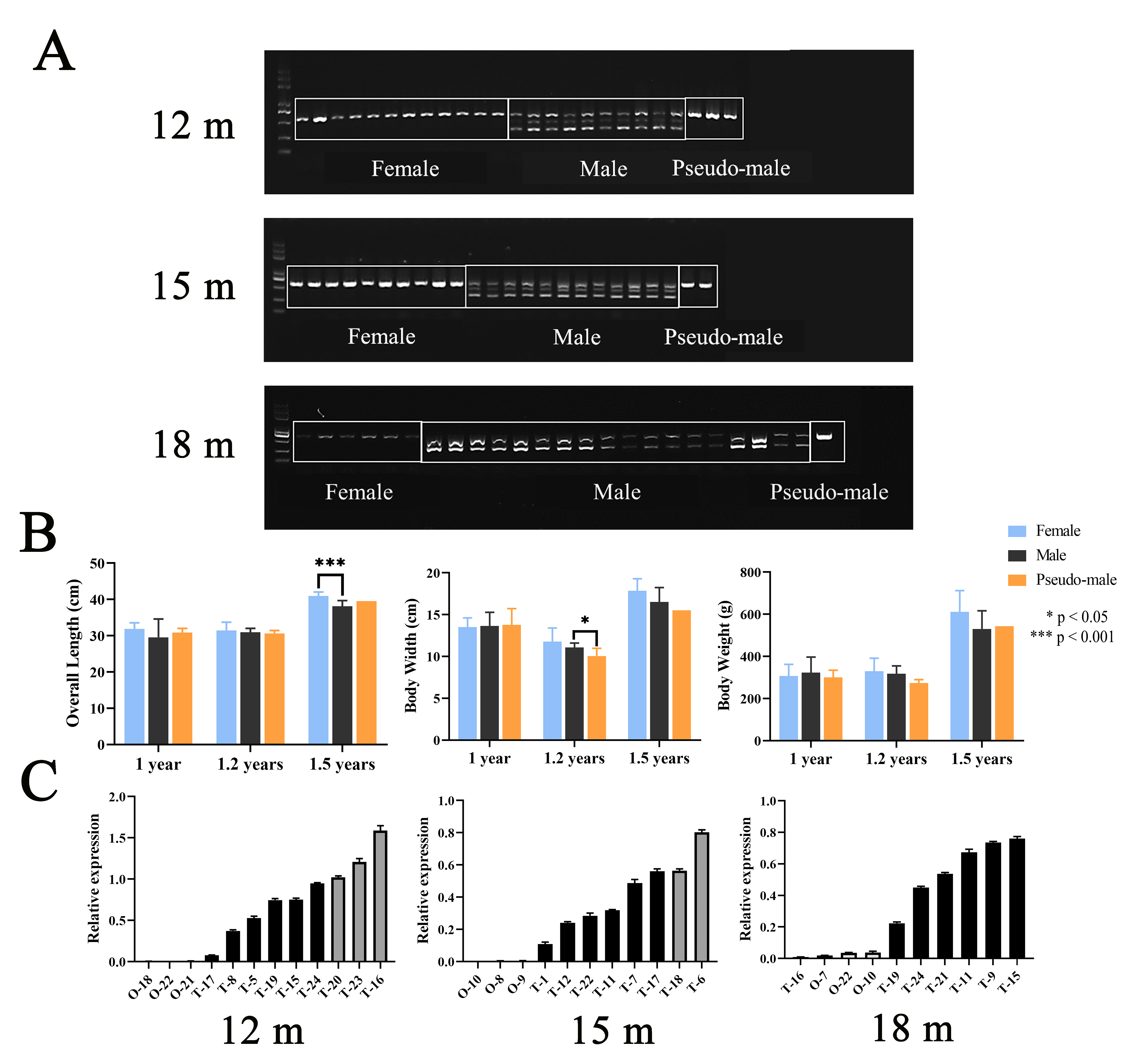

Supplement: Supplementary file 1 [file ijms-25-05935-s001.zip › Figure S1. Genetic sex-resize2.tif]

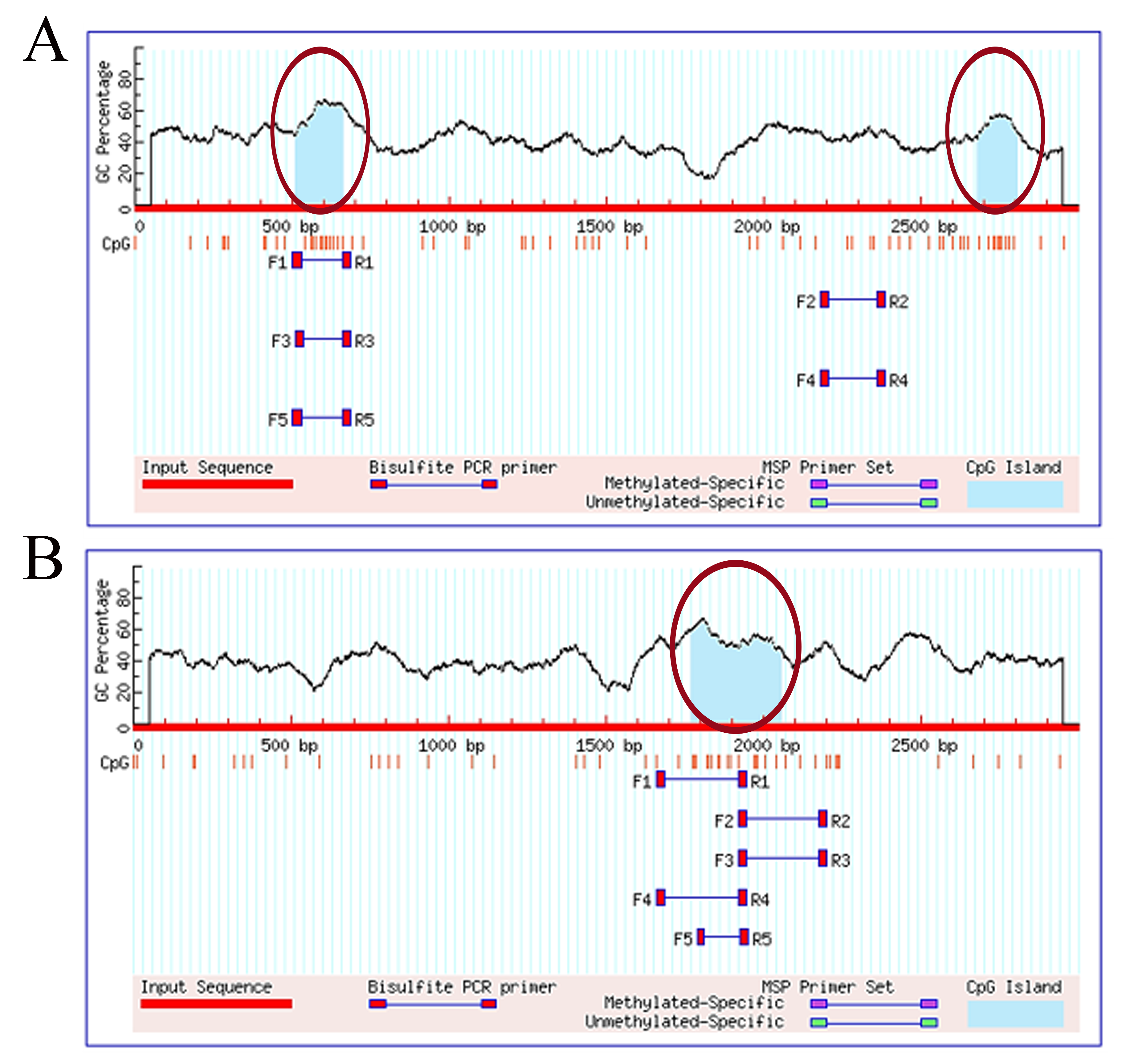

Supplement: Supplementary file 1 [file ijms-25-05935-s001.zip › Figure S2. CpG islands-resize.tif]

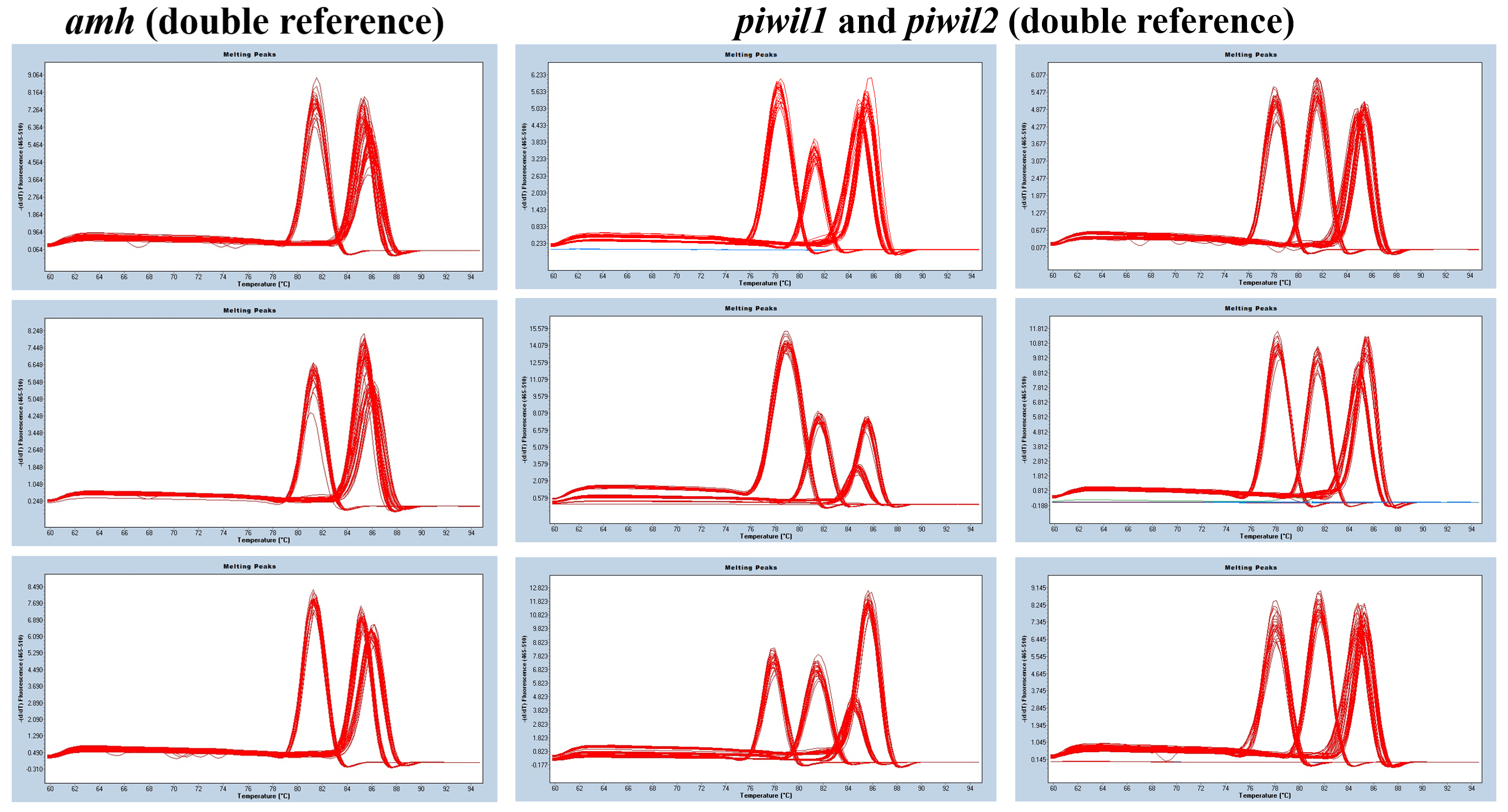

Supplement: Supplementary file 1 [file ijms-25-05935-s001.zip › Figure S3. Melting peaks.tif]
